# ST-elevation myocardial infarction caused by plaque erosion in a 23-year-old male

**DOI:** 10.1016/j.jccase.2025.06.007

**Published:** 2025-06-21

**Authors:** Genya Sunagawa, Daisuke Nagatomo, Keiji Oi, Shinki Nishioka, Masatsugu Nozoe, Koki Gondo, Nobuhiro Suematsu, Toru Kubota

**Affiliations:** a Division of Cardiology, Cardiovascular and Aortic Center, Saiseikai Fukuoka General Hospital, Fukuoka, Japan; b Department of Clinical Engineering, Saiseikai Fukuoka General Hospital, Fukuoka, Japan

**Keywords:** Non-ST-segment elevation myocardial infarction, Acute coronary syndrome, Plaque erosion, Spasm provocation

## Abstract

Intravascular imaging techniques, such as optical frequency domain imaging (OFDI), are essential for understanding the pathophysiology of acute coronary syndrome, including plaque rupture, plaque erosion, and calcified nodules. Plaque erosion is more common in younger patients than plaque rupture. We report a case of ST-elevation myocardial infarction caused by plaque erosion in a 23-year-old man. The patient presented with sudden-onset chest pain at work. Electrocardiography revealed ST-segment elevations in leads I, aVL, and V2–4. Coronary angiography identified thrombus formation in the left anterior descending artery (LAD) and total occlusion of the diagonal branch (D1). OFDI confirmed thrombus and plaque erosion in the LAD. Thrombus aspiration of the D1 restored thrombolysis in myocardial infarction grade 3 flow. Aspirated thrombus analysis revealed evidence of platelet aggregation and fibrin deposition. The absence of atherosclerosis or calcification on OFDI and a negative ergonovine provocation test supported the diagnosis of plaque erosion. Follow-up OFDI after three months showed thrombus resolution and residual fibrous plaque. This case highlights the role of OFDI in evaluating vascular characteristics during acute and chronic phases, enabling a precise diagnosis of plaque erosion.

**Learning objective:**

We report a case of ST-elevation myocardial infarction caused by plaque erosion in a 23-year-old patient. This case is noteworthy as it likely represents the first instance of thrombus originating from the left anterior descending artery and embolizing to the diagonal branch. The use of optical frequency domain imaging during both the acute and chronic phases allowed for precise assessment of the pathology.

## Introduction

Acute coronary syndrome (ACS) is primarily caused by plaque rupture, plaque erosion, and calcified nodules. Plaque rupture, accounting for 56–75 % of cases, is common in older individuals with comorbidities such as diabetes or dyslipidemia. In contrast, plaque erosion accounts for 20–44 % of ACS and is more common in young women [1,2]. It is also more commonly associated with non-ST elevation myocardial infarction (NSTEMI) than ST elevation myocardial infarction (STEMI).

Intravascular imaging techniques, including optical frequency domain imaging (OFDI) and optical coherence tomography (OCT), have become indispensable for investigating the pathophysiology of ACS, particularly STEMI. Plaque rupture results in disruption of the fibrous cap, establishing direct communication between the lumen and the necrotic core of an ulcerated, lipid-rich plaque. This process is associated with cavity formation and the development of a superimposed, red blood cell-rich thrombus, leading to significant luminal narrowing in OCT. In contrast, plaque erosion is characterized by an intact fibrous cap, irregular plaque surfaces, and thrombi adhering to the plaque surface without lipid core exposure or cavity formation [3]. The underlying plaque often appears fibrous and smooth.

Here, we present a case of STEMI caused by plaque erosion in a 23-year-old patient. OFDI was used in both phases to confirm diagnosis.

## Case report

The patient was a 23-year-old man with no past medical history of hypertension, dyslipidemia, diabetes mellitus, or developmental abnormalities. He smoked >30 cigarettes per day and worked as a laborer at a construction site. While at work, he experienced sudden-onset chest tightness that did not resolve with rest. He sought consultation with a nearby physician, where a test for heart-type fatty acid-binding protein was positive, prompting his transfer to our hospital.

**Fig. 1:**
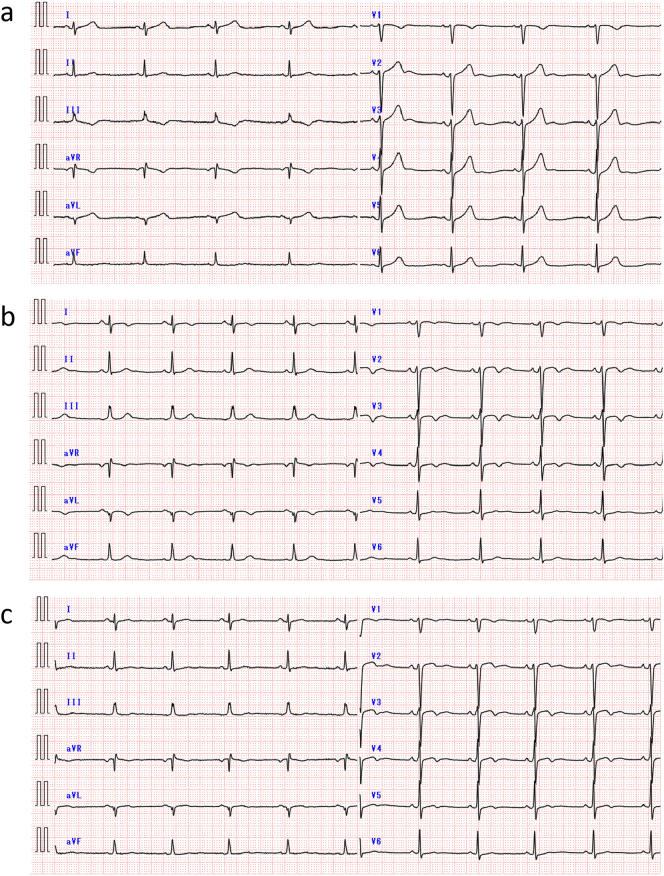
Serial 12-lead electrocardiograms. (a) On admission: ST-segment elevations in leads I, aVL, and V2–V4. (b) At 2 months: Resolution of ST-segment elevations with T-wave inversions and poor R wave progression in the precordial leads. (c) At 3 months: T-wave inversions appear shallower and R wave amplitudes slightly increase in leads V3–V4.

Upon arrival, the patient's vital signs were as follows: blood pressure of 126/78 mmHg, heart rate of 65 bpm (regular), and oxygen saturation of 100 % on room air. His height was 160 cm, weight was 70.7 kg, and body mass index was 27 kg/m^2^. Physical examination revealed no significant findings, although the chest tightness persisted. Electrocardiography (ECG) demonstrated sinus rhythm, ST-segment elevations in leads I, aVL, and V2–4 (Fig. 1a). A chest X-ray revealed a cardiothoracic ratio of 53 % with clear costophrenic angles. Transthoracic echocardiography showed mild anterior wall hypokinesis without valve disease or effusion. The left ventricular end-diastolic and end-systolic diameters were 51 mm and 32 mm, respectively, with interventricular septal and posterior wall thicknesses of 10 mm and 9 mm, and an ejection fraction of 67 %.

Laboratory findings revealed an elevated white blood cell count of 13,600/μL, aspartate aminotransferase of 22 U/L, alanine aminotransferase of 49 U/L, low-density lipoprotein cholesterol of 107 mg/dL, and hemoglobin A1c of 5.2 %. The high-sensitivity cardiac troponin T level was 0.095 ng/mL upon admission.

**Fig. 2:**
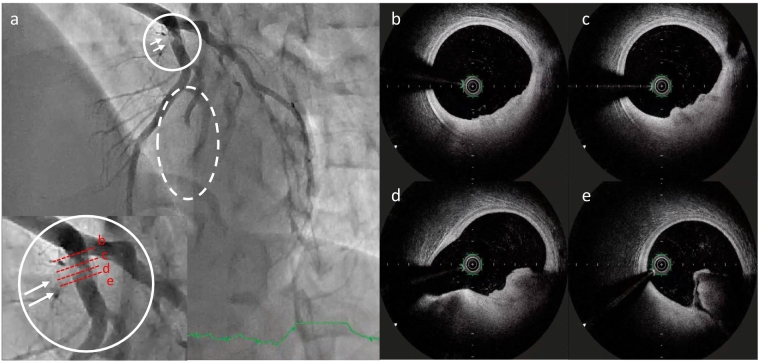
Angiography and optical frequency domain imaging findings on the day of ST elevation myocardial infarction. (a) Thrombus in the proximal left anterior descending artery (LAD) (in the white circle and indicated by the white arrow), showing a translucent appearance. Interruption of coronary flow in the first diagonal branch (in white dashed circle). (b–e) Optical frequency domain imaging images in LAD showing white thrombus formation on a fibrous cap observed between the 3 o'clock and 6 o'clock positions in four different frames (from proximal to distal, extending from the upper left to the lower right).

Emergent coronary angiography (CAG) was performed using a 6–7 Fr glide sheath via the right radial artery with an initial heparin bolus of 2000 units. CAG revealed total occlusion of the first diagonal branch (D1) and thrombus translucency in the proximal left anterior descending artery (LAD) (Fig. 2a). After administration of aspirin (200 mg), prasugrel (20 mg), and an additional 6000 units of intra-arterial heparin, percutaneous coronary intervention (PCI) was initiated. A Judkins left 3.5 guide catheter was used to engage the left coronary artery (LCA). A guidewire was successfully advanced to the distal LAD, and OFDI revealed a fibrous cap with residual white thrombus at the proximal LAD (Fig. 2b–e). A second wire was advanced to the distal D1, but flow was not restored. Thrombus aspiration was performed, and subsequent CAG demonstrated thrombolysis in myocardial infarction (TIMI) grade 3 flow in D1. Due to the patient's young age and lack of atherosclerosis, balloon angioplasty and stent implantation were avoided. Following PCI, his symptoms resolved completely. Histological analysis of the aspirated thrombus revealed platelet aggregation, fibrin deposition, and inflammatory cells, including erythrocytes and neutrophils, with no pathological evidence of atherosclerosis.

The troponin-T level peaked at 2.440 ng/mL two hour post-procedure, and creatine kinase peaked at 1494 U/L the following day. The patient recovered well and was discharged on day 7 with aspirin (100 mg daily) and prasugrel (100 mg daily).

**Fig. 3:**
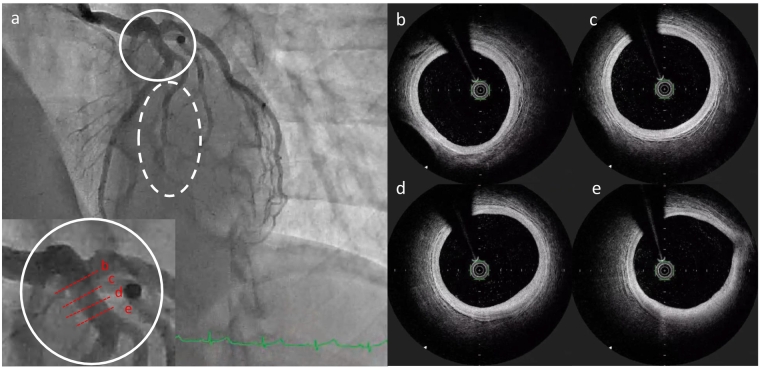
Angiography and optical frequency domain imaging (OFDI) findings three months post-treatment. (a) Resolution of the thrombus in the proximal left anterior descending artery (LAD), with thrombolysis in myocardial infarction grade 3 flow restored in diagonal branch. (b–e) OFDI images in LAD showing resolution of thrombus but still a little fibrous cap between the 3 o'clock and 5 o'clock positions in the upper right panel.

At the two-month follow-up, the ECG demonstrated deep T-wave inversions and loss of R wave progression in leads V1–V4 (Fig. 1b). At the three-month follow up, the ECG showed shallower T-wave inversions and partial recovery of R wave amplitude (Fig. 1c). CAG showed no stenosis in the coronary arteries (Fig. 3a). OFDI confirmed the absence of thrombus at the previous erosion site in the LAD (Fig. 3b–e). An ergonovine stress test was also performed, which did not induce coronary spasm. The patient has since been maintained on aspirin monotherapy and has experienced no recurrence of chest symptoms to date.

## Discussion

We report a rare case of STEMI in a 23-year-old man attributed to plaque erosion in the LAD with the D1. OFDI revealed a white thrombus in the LAD, while pathological examination confirmed similar findings in the D1 clot, strongly suggesting that thrombus dislodgement from the LAD caused the D1 occlusion.

OFDI enables longitudinal assessment of plaque erosion and thrombus resolution. In this case, OFDI identified fibrous caps and thrombus without evidence of atherosclerosis or calcification, consistent with plaque erosion. Follow-up OFDI three months later confirmed complete thrombus resolution. Epidemiological studies highlight that plaque erosion typically occurs at a younger age than plaque rupture or calcified nodules, with risk factors including female gender, age under 40 years, tobacco use, and absence of multi-vessel disease. Coronary spasm has been implicated in erosion cases, with Shin et al. [4] reporting its presence in over 25 % of cases. As we described, we performed the ergonovine provocation test at three-month follow up. Initially, CAG was performed for both the left and right coronary arteries without nitrate administration. Subsequently, intracoronary ergonovine was administered in two 20-μg doses into the right coronary artery, and in sequential 20- and 40-μg doses into the LCA. After each administration, the patient was monitored for 2 min for chest symptoms or ECG changes. CAG was then repeated to assess for any angiographic evidence of vasospasm. Throughout the procedure, no chest symptoms, electrocardiographic abnormalities, or angiographic changes were observed. Although the ergonovine provocation test was negative, the patient's heavy smoking history raises the possibility of vasospastic activity contributing to endothelial injury. A single negative test may not exclude vasospastic angina, particularly in heavy smokers [5]. Recent studies have highlighted a significant association between coronary artery spasm and plaque erosion. OCT analyses have demonstrated that plaque erosion is more prevalent in segments exhibiting vasospasm compared to non-spasm segments, suggesting that transient vasoconstriction may contribute to endothelial injury and subsequent thrombus formation [6]. Furthermore, prolonged vasospasm can cause mechanical stress and oxidative injury to the endothelium, promoting thrombosis and plaque erosion [7]. These findings suggest that in patients with plaque erosion, particularly those with risk factors such as heavy smoking or suspected vasospastic activity, therapeutic strategies targeting coronary spasm — including calcium channel blockers or aggressive risk factor modification such as smoking cessation — may be beneficial to prevent recurrent thrombotic events.

Plaque erosion usually causes NSTEMI, but rare STEMI from thrombus embolization has been reported [8]. In cases of plaque erosion, minor endothelial injury can induce localized platelet aggregation even in the absence of significant luminal narrowing. When antegrade blood flow is preserved, superficial platelet-rich thrombi may detach from the erosion site and embolize into distal branches [8]. Distal embolization in erosion has been described, but no report clearly shows embolization from LAD to D1. Therefore, our case may represent a uniquely documented instance of thrombus embolization from the LAD to the D1. Erosion typically results in localized, platelet-mediated thrombus formation with limited coagulation cascade involvement, causing partial obstruction rather than complete occlusion. In contrast, plaque rupture leads to large thrombi and total occlusion, typical of STEMI [9]. In our case, the embolized thrombus caused transient total occlusion of the D1 branch, causing STEMI despite limited LAD thrombus. The histological and imaging correlation strongly support embolic propagation from the LAD to the D1.

The EROSION study demonstrated that in selected patients with plaque erosion, thrombus aspiration followed by dual antiplatelet therapy - without stent implantation - was associated with good outcomes. Importantly, patients included in the study had no residual stenosis >70 %, no flow-limiting dissection, and good distal flow post-aspiration. Our patient met similar criteria: TIMI 3 flow, no severe narrowing, and young age without calcification or rupture, thus supporting the decision to avoid stenting [10].

Although OFDI was not performed in the D1, angiographic and histopathological findings provided sufficient evidence for distal embolization. We initially considered imaging the D1 with OFDI to confirm the presence of embolic thrombus. However, due to the restoration of TIMI 3 flow and a conservative institutional policy to avoid excessive manipulation in small-caliber vessels - especially in young patients - the decision was made not to proceed.

This case underscores the importance of recognizing plaque erosion as a potential mechanism for STEMI, even in young patients, and highlights OFDI's role in understanding disease in acute and chronic phases.

## Conclusion

We experienced a young STEMI case with plaque erosion confirmed by OFDI in the acute and chronic phase. We considered that erosion of the LAD led to thrombus migration to the diagonal branch, resulting in myocardial infarction.

## Patient permission/consent statement

Informed consent was obtained from the patient for the publication of the case and accompanying images.

## Declaration of Generative AI and AI-assisted technologies in the writing process

During the preparation of this work, the authors used ChatGPT, an AI language model developed by OpenAI, in order to assist with formatting and language refinement of the manuscript. After using this tool, the authors reviewed and edited the content as needed and take full responsibility for the content of the publication.

## Declaration of competing interest

The authors declare that there is no conflict of interest
